# Elevation of CXCL1 indicates poor prognosis and radioresistance by inducing mesenchymal transition in glioblastoma

**DOI:** 10.1111/cns.13297

**Published:** 2020-03-18

**Authors:** Wahafu Alafate, Xiaodong Li, Jie Zuo, Hua Zhang, Jianyang Xiang, Wei Wu, Wanfu Xie, Xiaobin Bai, Maode Wang, Jia Wang

**Affiliations:** ^1^ Department of Neurosurgery The First Affiliated Hospital of Xi'an Jiaotong University Xi'an China; ^2^ Center of Brain Science The First Affiliated Hospital of Xi'an Jiaotong University Xi'an China; ^3^ The Second Affiliated Hospital of Xi'an Jiaotong University Xi'an China

**Keywords:** CXCL1, epithelial‐mesenchymal transition, glioblastoma, NF‐κB, radioresistance

## Abstract

**Introduction:**

Glioblastoma (GBM) is identified as a lethal malignant tumor derived from the nervous system. Despite the standard clinical strategy including maximum surgical resection, temozolomide (TMZ) chemotherapy, and radiotherapy, the median survival of GBM patients remains <15 months. Accumulating evidence indicates that rapid‐acquired radioresistance is one of the most common reasons for GBM recurrence. Therefore, developing novel therapeutic targets for radioresistant GBM could yield long‐term cures.

**Aims:**

To investigate the functional role of CXCL1 in the acquired radioresistance and identify the molecular pathway correlated to CXCL1.

**Results:**

In this study, we identified that CXCL1 is highly expressed in GBM and the elevation of CXCL1 is involved in radioresistance and poor prognosis in GBM patients. Additionally, silencing CXCL1 attenuated the proliferation and radioresistance of GBM cells. Furthermore, we demonstrated that CXCL1‐overexpression induced radioresistance through mesenchymal transition of GBM via the activation of nuclear factor‐kappa B (NF‐κB) signaling.

**Conclusion:**

CXCL1 was highly enriched in GBM and positively correlated with poor prognosis in GBM patients. Additionally, elevated CXCL1 induced radioresistance in GBM through regulation of NF‐κB signaling by promoting mesenchymal transition in GBM.

## 1 INTRODUCTION

Glioblastoma (GBM) is one of the most prevalent and lethal tumors in the central nervous system with a severe median survival of <15 months.^1^ Even underwent standard clinical strategies including maximum surgical resection and concurrent chemo‐radiotherapy, overall survival of GBM patients has barely prolonged.^2^ Accumulating evidence showed that rapid‐acquired radioresistance is part of the major causes for failure of radiotherapy and recurrence of GBM.^3^ Therefore, developing novel therapeutic targets for adaptive therapeutic resistance of GBM is urgently needed.

Chemokines (chemotactic cytokines) are a set of small peptides regulating immune and inflammatory reactions in cells.^4^ Recent studies show that chemokines are critical for angiogenesis, hematopoiesis, tumorigenesis and metastasis and therapy resistance in cancer.^5^, ^6^, ^7^, ^8^ C‐X‐C Motif Ligand 1 (CXCL1) belongs to the chemotactic superfamily and is proven to be highly expressed in several types of cancers, including melanoma,^9^ gastric cancer,^10^ and bladder cancer.^11^ CXCL1 is reported to be significantly elevated in colorectal cancer and is closely associated with tumor size, depth of the invasion, grade, and overall survival.^12^, ^13^ Additionally, CXCL1 induces radioresistance by enhancing cellular DNA damage repair in a ROS‐dependent manner in esophageal squamous cell carcinoma.^14^ Moreover, paracrine CXCL1 drives gastric cancer cells invade into the lymphatic system through integrin β1/FAK/AKT signaling pathway.^15^ However, the potential correlation between CXCL1 and glioblastoma (GBM) progression still remains unclear.

It has been recognized that cells possess the ability to transition from epithelial to mesenchymal during embryonic development, by which cells modify their adhesion molecules to adopt a migratory or invasive behavior.^16^ Epithelial‐mesenchymal transition (EMT) is regarded as essential for migratory and invasive features, inducing stem cell properties, preventing apoptosis, and senescence, as well as contributing to immunosuppression and therapy resistance.^17^ Intriguingly, GBM cells utilize the ability to undergo mesenchymal transition, either spontaneously,^18^ or in response to radiotherapy.^19^

In this study, we identified that CXCL1 is highly enriched in GBM and the elevation of CXCL1 is associated with radioresistance and poor prognosis in GBM patients. Additionally, knockdown of CXCL1 attenuated the proliferation and enhanced radiosensitivity of GBM cells. Furthermore, we demonstrated that CXCL1 induced mesenchymal transition of GBM via activation of NF‐κB signaling.

## 2 MATERIALS AND METHODS

### 2.1 Gene expression analysis

Expression data of GSE56937 and GSE67089 were extracted from GEO database (McDonald et al^20^ and Mao et al^21^). These gene expression profile data were preprocessed by background correction, gene symbol transformation, and normalization through R programming. The limma package was then used for identifying differentially expressed genes (DEGs).^22^ The expression difference of individual gene was defined by log_2_FC (Fold change) and adjusted *P* value, in which log_2_FC > 1 with an adjusted *P* value < .05 was identified as an upregulated gene. Venn diagram was carried out to find the overlapping upregulated genes in different datasets.

### 2.2 Quantitative RT‐PCR (qRT‐PCR)

Total RNA was extracted using RNeasy mini kits according to the manufacturer's instruction as previously described.^23^ Concentration of RNA was carefully determined by Nanodrop 2000. GoldenstarTM RT6 cDNA Synthesis Kit (TsingKe Biotech) was used to synthesize cDNA strictly following the manufacturer's protocol. qRT‐PCR was then performed by using 2x T5 Fast qPCR Mix (TsingKe Biotech) on a StepOnePlus real‐time PCR system. GAPDH was used as an internal control. The sequences of the primers were shown as below:

NF‐κBforward, AACAGCAGATGGCCCATACC and reverse, AACCTTTGCTGGTCCCACAT; CDH1 forward, AGTGACTGATGCTGATGCCC and reverse, AATGTACTGCTGCTTGGCCT; CDH2 forward, GTGCATGAAGGACAGCCTCT and reverse, TGGAAAGCTTCTCACGGCAT; VIM forward, TCCGCACATTCGAGCAAAGA and reverse, TGATTCAAGTCTCAGCGGGC; β‐actin forward, CGGCGCCCTATAAAACCCA and reverse, CGCGGCGATATCATCATCCA. Relative mRNA expressions were calculated by 2‐ΔΔt method.

### 2.3 Western blot

Western blot was performed as previously described.^24^ Antibodies used in this study were shown as below: Anti‐CXCL1 primary antibody was purchased from Abcam (cat. no. ab86436). Anti‐β‐actin antibody was purchased from Abcam (cat. no. ab115777). Anti‐E‐cadherin, Anti‐N‐cadherin, and Anti‐Vimentin primary antibodies were purchased from CST (cat. no. #3195, #13116 and #5741, respectively). Anti‐Rabbit IgG and Anti‐Mouse‐IgG were purchased from CST (cat. no. #7074 and #7076, respectively).

### 2.4 Patient and glioma samples

This study was approved by the Scientific Ethics Committee at the First Affiliated Hospital of Xi'an Jiaotong University (approval no. 2016‐18). Ninety‐one glioma samples and three nontumor tissue samples (from epilepsy surgery) were collected from patients underwent surgical operations from 2008 to 2016. All the patients' samples have obtained necessary consent. The samples were embedded in paraffin blocks as previously described.^25^

### 2.5 Immunohistochemistry (IHC)

Immunohistochemistry was performed as previously described.^23^, ^24^, ^25^ Anti‐CXCL1 primary antibodies were purchased from Abcam (cat. no. ab86436). Goat anti‐rabbit IgG (cat. no. ab97051, Abcam) and goat anti‐mouse IgG (cat. no. ab205719, Abcam) were used as secondary antibodies. Tissues embedded with paraffin were cut into 4‐mm sections followed by deparaffinized, rehydrated, and stained with primary antibodies overnight at 4°C. Afterward, the slides were incubated with corresponding secondary antibodies and stained with DAB. At last, the slides were counterstained with hematoxylin and images were taken under the light microscope.

### 2.6 Lentivirus production and transduction

Lentivirus production and transduction was conducted as previously described.^24^ The plasmid for shCXCL1 lentivirus was designed and synthesized by Genepharma. shRNAs were designed and inserted into GV248 vector to construct stable cell lines. Stable clones transfected with shCXCL1 were selected for 4 weeks by puromycin. Targeting sequences were shown below:

- shCXCL1#1: CCGGCAAATGGCCAATGAGATCATTCTCGAGAATGATCTCATTGGCCATTTGTTTTTG
- shCXCL1#2: CCGGGTTCTCCAGTCATTATGTTAACTCGAGTTAACATAATGACTGGAGAACTTTTTG

The CXCL1‐overexpression lentivirus was designed by Genechem.

### 2.7 Cell culture and in vitro cell viability assay

Glioblastoma cell lines U251, SHG‐44, and NHA were provided by the First Affiliated Hospital of Xi'an Jiaotong University (Xi'an, China). U87 cell line was bought form BeNa Culture Collection. Cells were cultured in DMEM‐F12 containing 10% vol FBS and antibiotics (1% penicillin and streptomycin). All these cells were cultured in a humidified condition containing 5% CO_2_ at 37°C. As for cell viability assay, cell number was calculated by cell counter with trypan blue, then cells were seeded into 96‐well plates after adequate suspension at a density of 2 × 10^3^ cells/100 uL per well and cultured for 12 hours. After 24 hours of culturing at 37°C with 5% CO2, U87 and U251 cell lines received in vitro radiotherapy using X‐RAD 320 from Precision X‐ray at a dose of 8 Gy. Afterward, these cells were used to conduct indicated experiments. Finally, cell number was counted by alamarBlue and IC50 was calculated by using SPSS 22.0.

### 2.8 In vivo intracranial xenograft tumor models

The usage of experimental animals in this study was approved by the Ethics Committee of the School of Medicine, Xi'an Jiaotong University (approval no. 2016‑085). In vivo xenograft model was performed by using 6‐week‐old female nude mice. Corresponding GBM cell (pretransfected with shNT, shCXCL1#1 and shCXCL1#2) was suspended and diluted to the density of 1 × 10^5^ cells in 2 uL PBS then injected into the nude mice brains as described previously.^24^ Each group of treatment consists of five mice, and they were monitored unless the following symptoms came out: arched back, unsteady gait, more than 10% bodyweight loss, or leg paralysis.

### 2.9 Flow cytometry

Flow cytometry assays were conducted as previously described.^24^ Cell apoptosis was measured by the Alexa Fluor^®^ 488 Annexin V/Dead Cell Apoptosis kit strictly following the manufacturer's protocols.

### 2.10 Gene set enrichment analysis (GSEA) and Gene ontology (GO) analysis

Gene expression profiles were derived from the Cancer Genome Atlas (TCGA) database. All data were preprocessed by background correction, gene ID transformation, and normalization by R. Furthermore, these data were ordered by the expression of CXCL1 to divide all samples into two groups, CXCL1^high^ and CXCL1^low^ (by quartile cutoff). Subsequently, limma package^22^ was used to calculate the differentially expressed genes in these two groups. Afterward, gene ontology (GO) annotation was conducted using the Database for Annotation, Visualization and Integrated Discovery online tool (DAVID, https://david.ncifcrf.gov/).

As for gene set enrichment analysis (GSEA), the GSEA software^26^ was carried out to elucidate the essential pathways that are significantly enriched in CXCL1^high^ groups.

### 2.11 Statistical analysis

All the results in this study are exhibited as mean ± SD (Standard Deviation). Number of independent replications is presented in the corresponding Figure legends. Two‐tailed *t* tests were used to evaluate statistical differences. One‐way ANOVA was utilized in comparisons more than two groups, following Dunnett's post‐test. Kaplan‐Meier survival analysis was conducted using log‐rank analysis. All statistical analysis was calculated by GraphPad Prism 7 or SPSS 22.0 software. Unless specifically indicated, statistical significance was considered as a two‐sided *P* value < .05.

## 3 RESULTS

### 3.1 CXCL1 is significantly upregulated in radiotherapy and mesenchymal subtype

**Figure 1:**
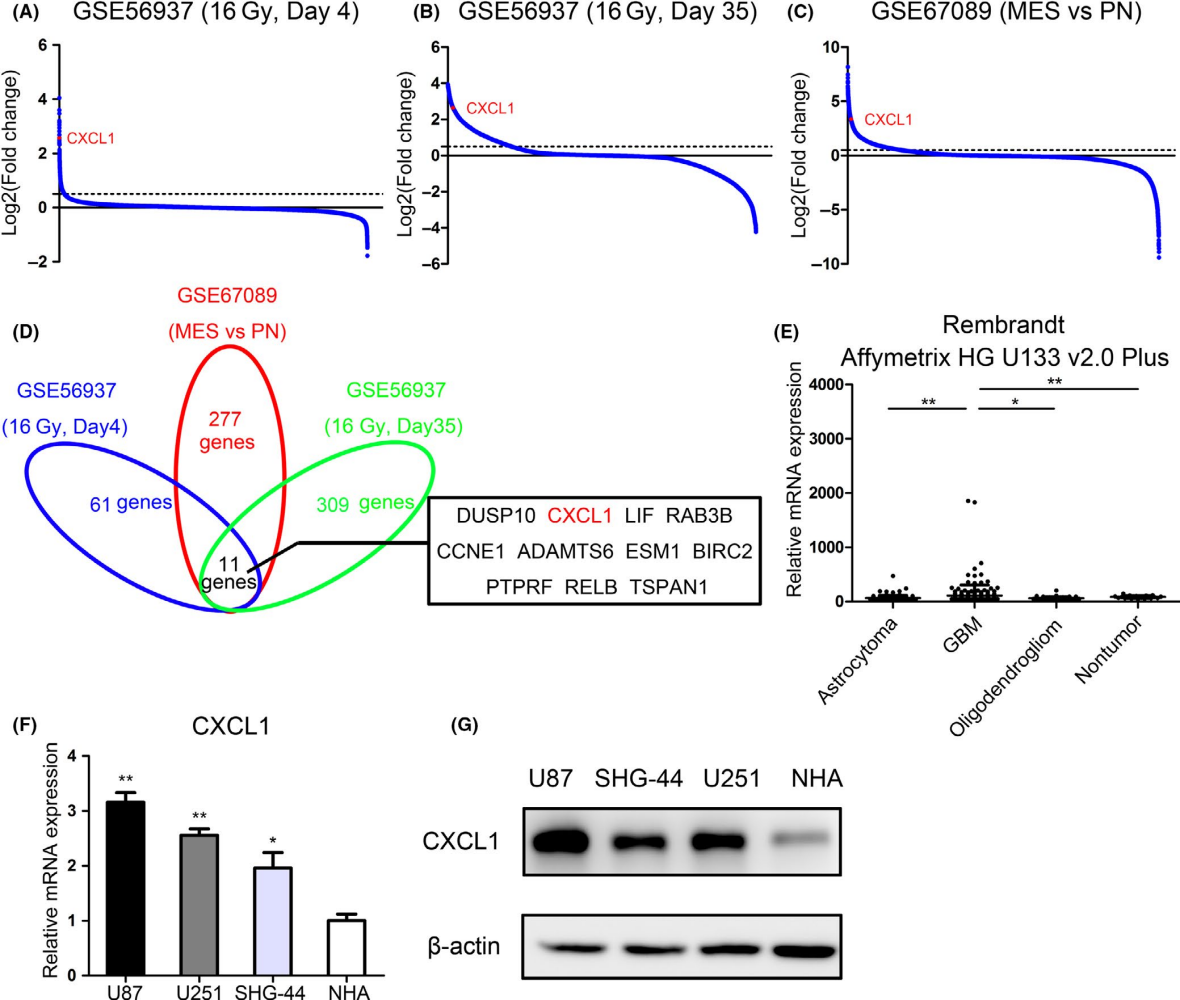
CXCL1 is significantly upregulated in radiotherapy and mesenchymal subtype. A, Microarray analysis for differentially expressed genes (DEGs) in short‐term radiotherapy (16Gy, 4 d vs negative control) using GEO database (GSE56937). B, Microarray analysis for DEGs in long‐term radiotherapy (16Gy, 35 d vs negative control) using GEO database (GSE56937). C, Microarray analysis for DEGs in mesenchymal subtype of GBM cells (MES vs PN) by calculating GEO database (GSE67089). D, Venn diagram for upregulated genes in both three sets of comparisons, indicating that CXCL1 was one of the most upregulated genes in radiotherapy as well as mesenchymal subtype. E, Gene expression analysis with Rembrandt database showed that CXCL1 was up‐regulated in GBM samples compared with Astrocytoma, Oligodendroglioma and nontumor tissues (*P < .05, **P < .01, with one‐way ANOVA followed by Dunnett's posttest). F, CXCL1 mRNA expression in human glioma cell lines and NHA was measured by qRT‐PCR (**P < .01, *P < .05, with one‐way ANOVA followed by Dunnett's post‐test). G, CXCL1 protein expression in human glioma cell lines and NHA was measured by Western blot analysis. β‐actin was used as an internal control. All data were reported as the mean ± SD of triplicate independent experiments

It is well known that mesenchymal GBM represents more aggressive and invasive signatures including rapid progression, recurrence, and radioresistance.^27^, ^28^ To identify candidate genes correlated to radioresistance, hierarchical bi‐clustering was performed by using previously published GEO databases (GSE56937 and GSE67089). The differentially expressed genes (DEGs) in these databases were classified by fold changes (Figure 1A‐C). As is illustrated in Figure 1D, Venn diagram was drawn to find out the overlapping upregulated genes. Totally, 11 genes were positively correlated with both rapid reaction and delayed reaction to radiotherapy as well as mesenchymal subtype, including DUSP10, CXCL1, LIF, RAB3B, CCNE1, ADAMTS6, ESM1, BIRC2, PTPRF, RELB, and TSPAN1. Moreover, CXCL1 was significantly upregulated in GBM according to Rembrandt database (Figure 1E). To investigate the clinical relevance, survival analysis was performed among the 11 gene candidates. The results indicated that only CXCL1(*P* = .016, with log‐rank test) and ESM1(*P* = .022, with log‐rank test) were significantly correlated to overall survival in GBM patients (Figure S1). Therefore, we choose CXCL1 as a candidate gene in this study. Afterward, U87 and U251 cell lines were chosen for the following experiments, which have a higher endogenous CXCL1 expression level (Figure 1F and G).

### 3.2 Elevated CXCL1 expression was closely associated with poor prognosis in glioma/GBM patients

**Figure 2:**
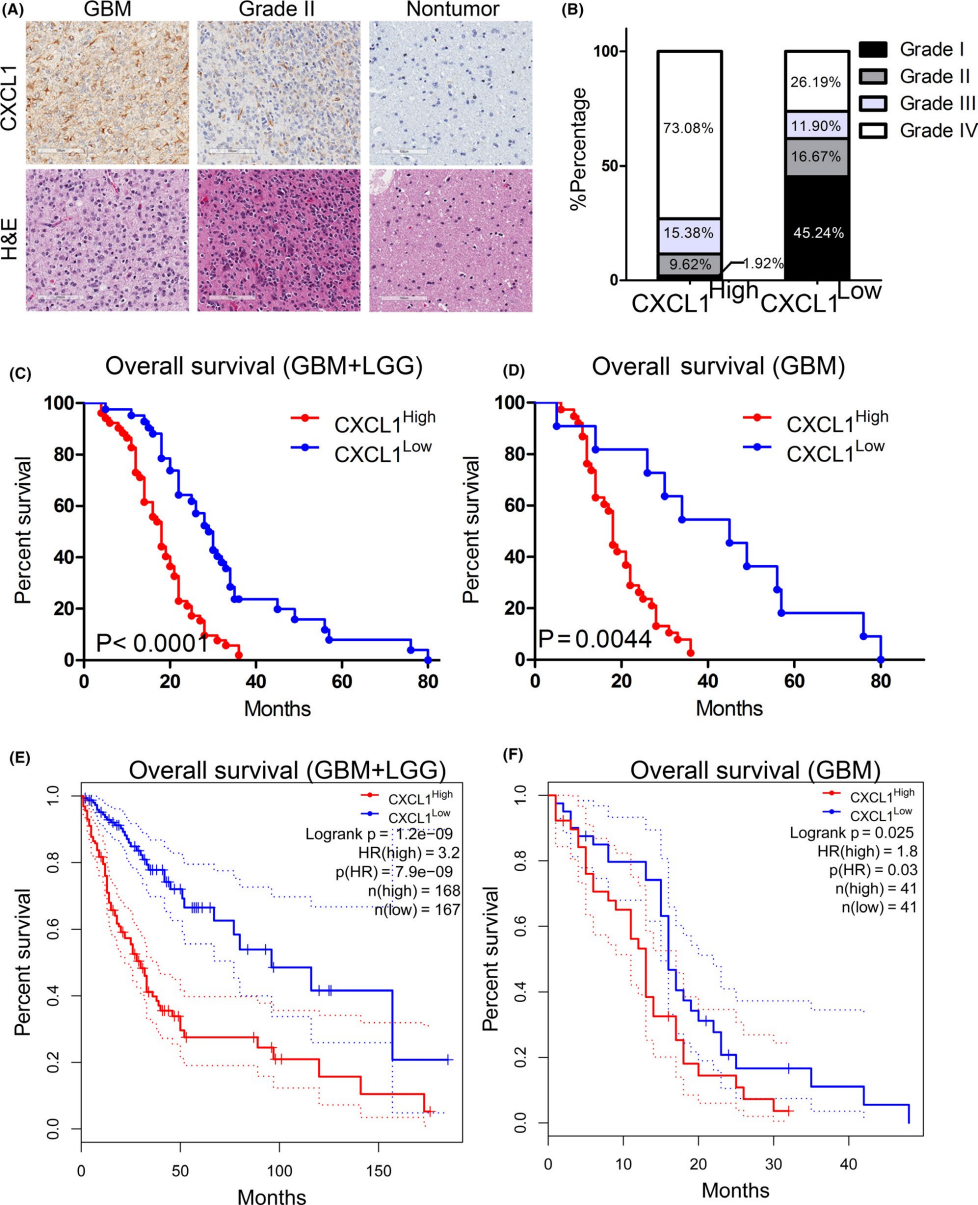
Elevated CXCL1 expression was closely associated with poor prognosis in glioma/GBM patients. A, Representative IHC images of CXCL1 in glioma samples. Upper panel: CXCL1 staining; Lower panel: H & E staining. Brain tissue from epilepsy surgery was used as negative controls. B, CXCL1 was enriched in high‐grade glioma samples. CXCL1^high^ samples accounted for 73.08% of GBM, while in CXCL1^low^ glioma samples, GBM accounted for 26.19%. C, Kaplan‐Meier analysis for CXCL1 expression with LGG and GBM patient samples (*P* < .0001, with log‐rank test). D, Kaplan‐Meier analysis for CXCL1 using GBM patient samples only (*P* = .0044, with log‐rank test). E, Kaplan‐Meier analysis for CXCL1 expression using TCGA GBM and LGG databases (*P* < .01, with log‐rank test). F, Kaplan‐Meier analysis with TCGA GBM database for CXCL1 expression (*P* = .025, with log‐rank test)

To build greater insight into the clinical significance of CXCL1, IHC staining was performed by using samples derived from patients underwent surgical resection in the Department of Neurosurgery, The First Affiliated Hospital of Xi'an Jiaotong University from 2009 to 2015. The results indicated that CXCL1 was markedly overexpressed in GBM compared with low‐grade glioma (Figure 2A and B). Moreover, Kaplan‐Meier analysis showed that increased CXCL1 expression was closely correlated to more severe overall survival than the lower ones (Figure 2C and D). Additionally, similar results could be seen in TCGA databases (Figure 2E and F). These data indicated that upregulation of CXCL1 revealed poor prognosis of glioma patients, especially in GBM, suggesting that CXCL1 could be considered as a potential diagnostic and prognostic biomarker for glioma and GBM.

### 3.3 Silencing CXCL1 attenuated the malignancy of glioma cell lines

**Figure 3:**
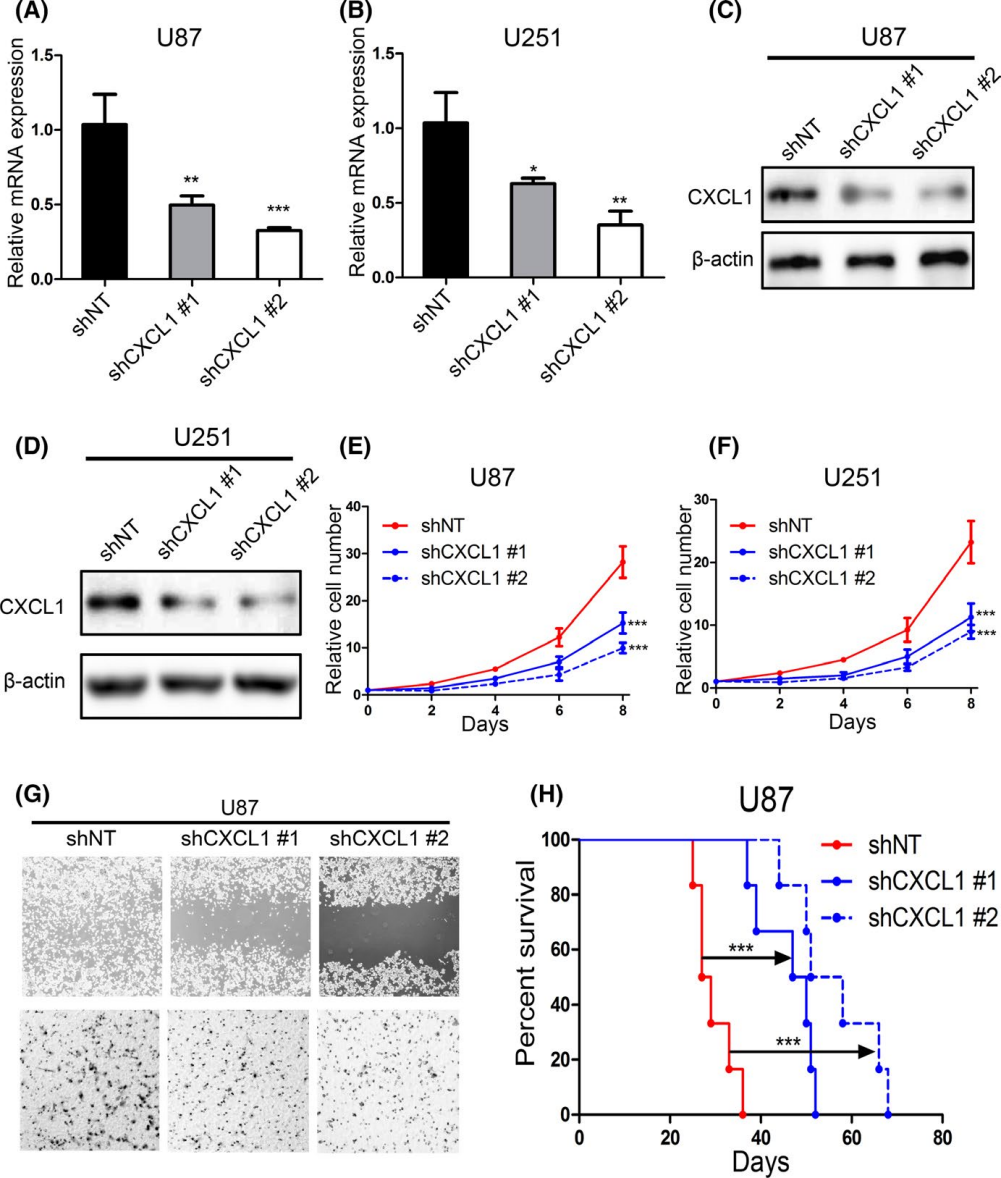
Silencing CXCL1 attenuated the malignancy of glioma cell lines. A and B, qRT‐PCR analysis for measuring the mRNA expression of CXCL1 in U87 and U251 cells transfected with lentiviral shCXCL1#1, shCXCL1#2, and negative control. C and D, Western blot analysis for detecting the CXCL1 protein expression in U87 and U251 cells transfected with lentiviral shCXCL1#1, shCXCL1#2, and negative control. E and F, Time survival curve of U87 and U251 cells transfected with lentiviral shCXCL1#1, shCXCL1#2, and negative control (***P < .001, with one‐way ANOVA followed by Dunnett's post‐test) G, upper panel: Wound healing assays were performed to determine the inhibition of migratory ability in shCXCL1 transfected cell lines. Lower panel: Matrigel invasion assays were used to identify the inhibition of invasive ability of CXCL1 silencing in shCXCL1 transfected cell lines. H, Kaplan‐Meier analysis for in vivo intracranial xenograft mice using U87 cells pretransfected with shCXCL1#1, shCXCL2#2, and negative control (***P < .001, with log‐rank test)

To further study the function of CXCL1 in GBM, 2 shRNAs targeting CXCL1 (shCXCL1 #1 and shCXCL1 #2) were introduced into U87 and U251 cells. qRT‐PCR analysis illustrated that the CXCL1 mRNA expression was reduced more than 50% in both U87 and U251 transfected with shCXCL1 lentivirus compared with shNT cells (Figure 3A and B). Consistently, protein levels of CXCL1 were also significantly reduced by exogenous lentiviral knockdown (Figure 3C and D). Moreover, in vitro cell viability assays were carried out to explore the effect of CXCL1 knockdown on tumor proliferation of U87 and U251 cells. CXCL1 silencing markedly decreased the in vitro cell growth of U87 and U251 cells (Figure 3E and F). Furthermore, wound healing assays and matrigel invasion assays were utilized to evaluate the functional role of CXCL1 on migration and invasion of GBM. The results showed that both the closure time and the number of invasive cells were reduced in CXCL1‐knockdown U87 and U251 cells compared with the control cells (Figure 3G). In addition, Kaplan‐Meier analysis for in vivo xenograft mice models using U87 cells transfected with lentiviral shCXCL1 or shNT showed that shCXCL1 prolonged the survival time of xenograft mice. Altogether, our results illustrated that suppression of CXCL1 attenuated the proliferation, migration, and invasion of GBM cells both in vitro and in vivo*.*

### 3.4 CXCL1 overexpression enhanced radioresistance in GBM

**Figure 4:**
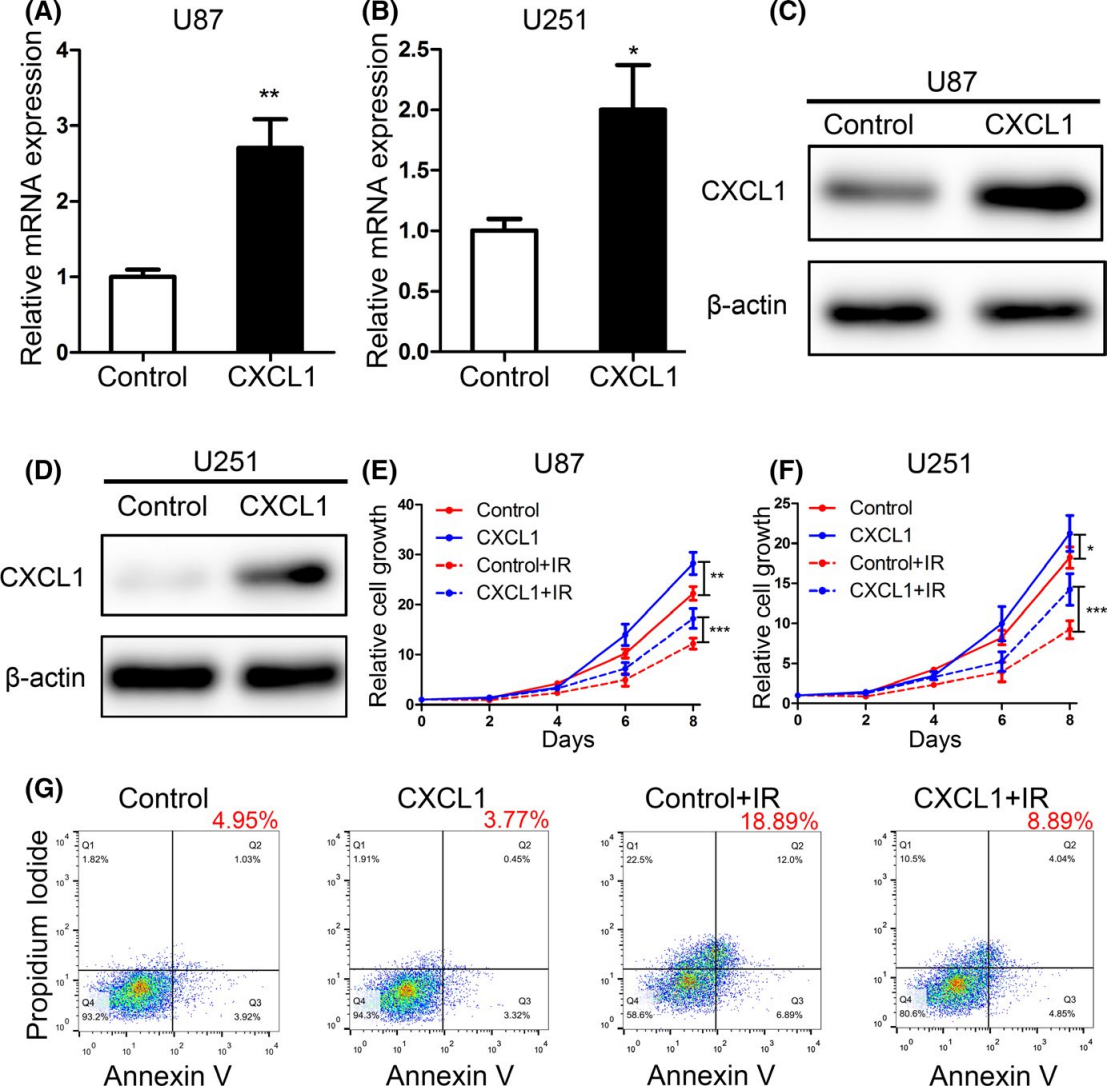
CXCL1 overexpression enhanced radioresistance in GBM cell lines. A and B, qRT‐PCR analysis was performed to determine the overexpression efficiency of CXCL1 in U87 and U251 cell lines (**P < .01, with *t* test). C and D, The overexpression of CXCL1 protein was detected by Western blot in U87 and U251 transfected with CXCL1‐overexpression lentivirus. E and F, In vitro cell proliferation assays were performed by using different interventions as indicated in U87 and U251 cell lines. (IR, ionizing radiation; ***P < .001, *P < .05, with one‐way ANOVA followed by Dunnett's post‐test). G, Flow cytometry analysis using Annexin V and propidium iodide for apoptotic ratio analysis in U87 and U251 cells pretreated with indicated interventions

To further verify the correlation between CXCL1 and radioresistance, CXCL1 was overexpressed in U87 and U251 cell lines via lentivirus infection. Afterward, the transfection efficiency was monitored by qRT‐PCR assays (Figure 4A and B) as well as Western blot (Figure 4C and D). Additionally, we combined CXCL1 overexpression with 8Gy radiation. As a result, CXCL1 overexpression promoted cell proliferation and enhanced radioresistance in U87 and U251 cells (Figure 4E and F). Moreover, flow cytometry indicated that the percentage of GBM cells underwent early (AV^+^; PI^‐^) and late (AV^+^; PI^+^) apoptosis were significantly diminished by CXCL1 overexpression followed by radiation, compared with radiotherapy alone (Figure 4G). Therefore, these data indicated that elevated CXCL1 promoted radioresistance in GBM.

### 3.5 CXCL1 induced radioresistance in GBM via regulation of NF‐κB signaling and mesenchymal transition

**Figure 5:**
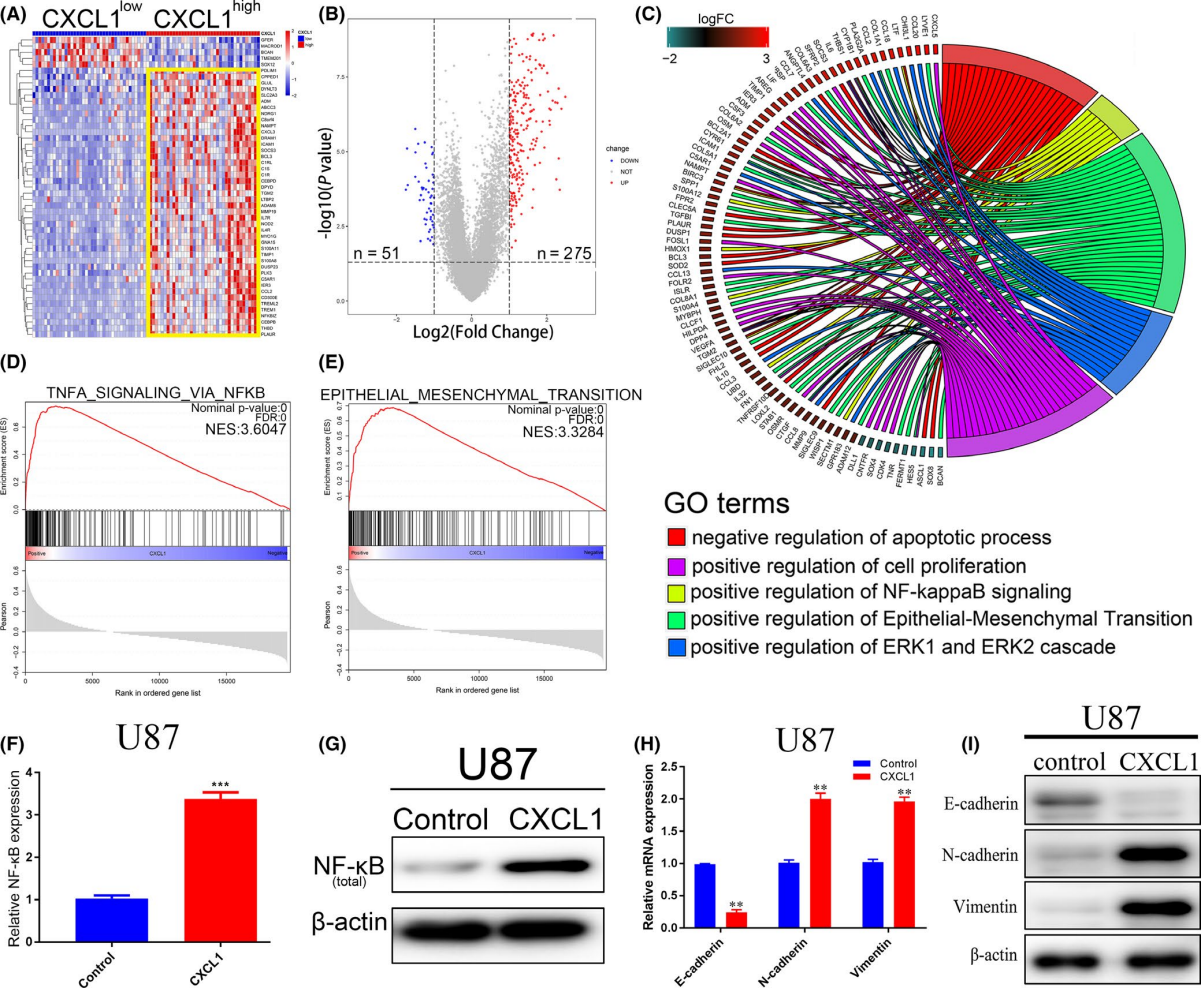
CXCL1 induced radioresistance in GBM via regulation of NF‐κB signaling and mesenchymal transition. A, Hierarchical bi‐clustering analysis was performed by using TCGA GBM database, indicating the significant gene signature in CXCL1^high^ GBM compared with CXCL1^low^ GBM. B, The amount of differentially expressed genes in CXCL1^high^ and CXCL1^low^ groups was presented by volcano plot (log_2_FC > 1 or log_2_FC<−1, with an adjusted *P* value < .05). C, Gene ontology (GO) analysis for differentially expressed genes. (CXCL1^high^ vs CXCL1^low^).D and E, Gene set enrichment analysis (GSEA) was performed to find out the highly enriched signaling pathways in CXCL1^high^ group. F, mRNA expression of NF‐κB was measured by qRT‐PCR analysis (***P < .001, with *t* test). G, Western blot of total NF‐κB/p65 expression, β‐actin was used as a loading control. H, mRNA expression of mesenchymal transition markers was measured by qRT‐PCR analysis (**P < .05, with one‐way ANOVA followed by Dunnett's post‐test). I, Western blot of mesenchymal transition markers. β‐actin was used as a loading control

As we showed that CXCL1 functions as an oncogene and induces radioresistance in GBM, bioinformatics analysis of TCGA database was performed to further evaluate the underlying signaling pathways for CXCL1. Patient samples from TCGA database were grouped into two groups according to the expression of CXCL1. Hierarchical bi‐clustering analysis indicated significant gene signatures in CXCL1^high^ GBM compared with CXCL1^low^ GBM (Figure 5A). Through grouping the TCGA GBM samples with a quartile cutoff, we demonstrated that 275 significantly upregulated genes in CXCL1^high^ group (log_2_FC > 1, adjusted *P* < .05) while 51 downregulated in CXCL1^low^ group (log_2_FC<−1, adjusted *P* < .05; Figure 5B). Moreover, to obtain more insight into these candidate pathways, Database for Annotation, Visualization and Integrated Discovery online tool (David, https://david.ncifcrf.gov/) was utilized to conduct the Gene Ontology (GO) annotation. Negative regulation of apoptotic process, positive regulation of cell proliferation, NF‐κB signaling, EMT, and ERK1/ERK2 cascade were the most enriched cancer‐related GO terms (Figure 5C). Additionally, GSEA results demonstrated that CXCL1 was associated with multiple molecular pathways related to malignancy and therapy resistance, including epithelial‐mesenchymal transition^29^ and NF‐κB signaling^30^ (Figure 5D and E).To further confirm the results of bioinformatics analysis, qRT‐PCR and Western blot assays were carried out. The results showed that CXCL1 overexpression significantly enhanced the overall expression of NF‐κB/p65, indicating that NF‐κB signaling might be activated by CXCL1. It is well known that NF‐κB is one of the mediators of EMT response, activated NF‐κB functions to regulate a range of transcription factors in order to manage the overall mesenchymal transition program.^31^ Therefore, qRT‐PCR and Western blot analyses were performed to confirm the correlation between NF‐κB and mesenchymal transition. Intriguingly, both mRNA and protein of mesenchymal transition markers were regulated by CXCL1 overexpression. Taken together, the results indicated that CXCL1 overexpression enhanced the mesenchymal signature of GBM via regulation of NF‐κB signaling.

## 4 DISCUSSION

Glioblastoma is one of the most lethal tumors in human central nervous system. Despite the development of clinical strategies during the past several decades, outcomes of GBM patient is still remains dismal.^32^ Acquired radioresistance characterizes a major cause of poor prognosis thus promotes tumor recurrence in GBM.^33^ Various molecular pathways have been proved to be responsible for the acquisition of radioresistance, including EMT,^19^ NF‐κB,^34^ and Wnt/β‐catenin.^35^ Accumulating data showed that CXCL1, a small cytokine expressed by macrophages, epithelial cells, and neutrophils,^36^, ^37^ was associated with therapy resistance in diverse subtypes of cancers.^8^, ^14^ Additionally, CXCL1 has also been shown to contribute to radioresistance through inducing DNA damage repair in a ROS‐dependent manner in esophageal squamous cell carcinoma, indicating that CXCL1 might be a potential target for suppressing radioresistance in cancer therapy.^14^ Moreover, it has been noted that CXCL1 promotes tumorigenesis, progression, and recurrence in a range of cancers including bladder cancer, pancreatic cancer, and glioma.^11^, ^38^, ^39^ However, the functional role of CXCL1 in GBM radioresistance has not been fully elucidated.

DNA damage repair is regarded as a crucial process for GBM cells to acquire radioresistance.^33^ Zhang et al^14^ reported that cancer‐associated fibroblast (CAF)‐secreted CXCL1 promoted radioresistance in a ROS‐dependent manner by regulating DNA damage repair. Additionally, irradiation or chemotherapy elevated NF‐κB activity and was correlated with therapy resistance and reduced apoptosis.^40^ When exposed to irradiation, cancer cells produce endogenous TNFα, which is proved to be a trigger for NF‐κB activation.^41^, ^42^ Furthermore, it has been well understood that the main detector of DNA damage includes the PI3 kinases ATM, ATR, and DNA‐PK, which phosphorylate multiple proteins and lead to DNA damage response.^43^ The serine/threonine protein kinase ATM is shown to recruit to DNA double‐strand break (DSB) site and active itself by phosphorylation, which leads to the catalytic active form of ATM dimers.^44^ ATM further causes activation of NF‐κB, which results in cell cycle regulation and DNA repair.^45^ Altogether, these reports suggest that NF‐κB is one of the key regulators of radioresistance and DNA damage repair, and downregulation of NF‐κB could serve as a new therapeutic targeting.

Recent studies have clarified that GBMs are highly plastic and have a natural tendency to transition from one subtype to another.^21^, ^27^ This transcriptional plasticity enables GBM to rapidly gain resistance to radiotherapy.^30^ Intrinsic therapy resistance has been caused by the process of mesenchymal transition, by which GBM cells acquired mesenchymal properties through loss of cell‐cell adhesion, acquisition of invasive, and migratory capability.^46^ It has been well studied that mesenchymal transition is responsible for oncology as well as invasion, metastasis, and therapy resistance of cancer cells.^47^ Recent studies have demonstrated that the in vitro survival of mesenchymal cells followed by high‐dose irradiation is significantly higher than those did not undergo EMT.^48^ Moreover, when exposed to fractionated irradiation at single doses less than 2Gy, indicating that mesenchymal GBM is more flexible to gain resistance to radiotherapy.^49^, ^50^ Mesenchymal transition is usually controlled by specific transcriptional factors like ZEB1 and SNAIL.^16^ ZEB1 and ZEB2 belong to the ZEB family of transcription factors, and both contain κB‐sites in their promoters,^51^ therefore, NF‐κB mediates the expression of ZEB mRNA by binding to their promoter and controls mesenchymal transition in GBM.^52^ Moreover, the protein SNAIL, which is also induced by NF‐κB, downregulates epithelial genes by binding to the promoter regions of E‐box sequences.^46^ These findings indicate that NF‐κB regulates and interacts with many master transcription factors which result in mesenchymal transition. Given that the activation of NF‐κB signaling induces more aggressive phenotype in GBM, inhibition of this signaling has been placed into corporate GBM treatment; however, there has been no apparent success.^31^

In this study, we analyzed the transcriptome expression profiles of two published GEO databases related to radioresistance and molecular subtype classification of GBM. As a result, CXCL1 was identified as one of the most upregulated genes in both radioresistant cells as well as mesenchymal cells. Next, elevated CXCL1 expression was closely related to poor prognosis in glioma/GBM patients. Furthermore, molecular biological assays were conducted to demonstrate the potential role of CXCL1 by lentiviral silencing and overexpression, and we illustrated that CXCL1 promotes radioresistance of GBM while suppressing CXCL1 attenuated the migratory and invasive behaviors of GBM cells. Moreover, in vivo tumor xenograft mice model demonstrated that CXCL1‐knockdown prolonged the overall survival of tumor‐xenografted mice and increased the sensitivity of GBM. Additionally, EMT and NF‐κB signaling were proved to be the most correlated pathways of CXCL1 expression according to the results of GSEA by using TCGA GBM database, which was also confirmed by molecular biological assays. Overall, our findings suggest that the elevation of CXCL1 indicates a poor prognosis and radioresistance by inducing mesenchymal transition in glioblastoma.

Although the potential role of CXCL1 in GBM radioresistance is well discussed in this study, additional researches on the molecular mechanism are still required for evaluating the clinical significance of CXCL1 inhibition in radiotherapy. Moreover, whether CXCL1‐induced radioresistance solely depends on its regulation in NF‐κB signaling and mesenchymal transition remains unclear. As GBM is a type of heterogeneous disease, inhibition of a single candidate biomarker might have unpredictable consequences. Therefore, in future studies, it will be critical to improve the overall management of GBM patients by focusing on specific subgroups of patients.

## 5 CONCLUSION

In conclusion, our findings suggest that CXCL1 is overexpressed in GBM and confers aggressive radioresistance to GBM cells, and suggest CXCL1 as a valuable biomarker for GBM patients. Both bioinformatic and functional analysis confirmed the role of CXCL1 in promoting radioresistance in GBM. We further identified EMT and NF‐κB as the downstream signaling pathways of CXCL1 and silencing CXCL1 attenuated tumor progression.

## CONFLICT OF INTEREST

The authors declare no conflict of interest.

## References

[1] Cui H , Zhang M , Wang Y , Wang Y . NF‐YC in glioma cell proliferation and tumor growth and its role as an independent predictor of patient survival. Neurosci Lett. 2016;631:40‐49. 27495011 10.1016/j.neulet.2016.08.003

[2] Chinot OL , Wick W , Mason W , et al. Bevacizumab plus radiotherapy‐temozolomide for newly diagnosed glioblastoma. N Engl J Med. 2014;370(8):709‐722. 24552318 10.1056/NEJMoa1308345

[3] Lee Y , Kim KH , Kim DG , et al. FoxM1 promotes stemness and radio‐resistance of glioblastoma by regulating the master stem cell regulator Sox2. PLoS One. 2015;10(10):e0137703. 26444992 10.1371/journal.pone.0137703 PMC4596841

[4] Schall TJ , Bacon KB . Chemokines, leukocyte trafficking, and inflammation. Curr Opin Immunol. 1994;6(6):865‐873. 7710711 10.1016/0952-7915(94)90006-x

[5] Cyster JG . Chemokines and cell migration in secondary lymphoid organs. Science. 1999;286(5447):2098‐2102. 10617422 10.1126/science.286.5447.2098

[6] Belperio JA , Keane MP , Arenberg DA , et al. CXC chemokines in angiogenesis. J Leukoc Biol. 2000;68(1):1‐8. 10914483

[7] Sallusto F , Mackay CR , Lanzavecchia A . The role of chemokine receptors in primary, effector, and memory immune responses. Annu Rev Immunol. 2000;18:593‐620. 10837070 10.1146/annurev.immunol.18.1.593

[8] Acharyya S , Oskarsson T , Vanharanta S , et al. A CXCL1 paracrine network links cancer chemoresistance and metastasis. Cell. 2012;150(1):165‐178. 22770218 10.1016/j.cell.2012.04.042 PMC3528019

[9] Dhawan P , Richmond A . Role of CXCL1 in tumorigenesis of melanoma. J Leukoc Biol. 2002;72(1):9‐18. 12101257 PMC2668262

[10] Wang L , Zhang C , Xu J , et al. CXCL1 gene silencing inhibits HGC803 cell migration and invasion and acts as an independent prognostic factor for poor survival in gastric cancer. Mol Med Rep. 2016;14(5):4673‐4679. 27748927 10.3892/mmr.2016.5843 PMC5102040

[11] Miyake M , Hori S , Morizawa Y , et al. CXCL1‐mediated interaction of cancer cells with tumor‐associated macrophages and cancer‐associated fibroblasts promotes tumor progression in human bladder cancer. Neoplasia. 2016;18(10):636‐646. 27690238 10.1016/j.neo.2016.08.002 PMC5043399

[12] Ogata H , Sekikawa A , Yamagishi H , et al. GROalpha promotes invasion of colorectal cancer cells. Oncol Rep. 2010;24(6):1479‐1486. 21042742

[13] Wang D , Dubois RN , Richmond A . The role of chemokines in intestinal inflammation and cancer. Curr Opin Pharmacol. 2009;9(6):688‐696. 19734090 10.1016/j.coph.2009.08.003 PMC2787713

[14] Zhang H , Yue J , Jiang Z , et al. CAF‐secreted CXCL1 conferred radioresistance by regulating DNA damage response in a ROS‐dependent manner in esophageal squamous cell carcinoma. Cell Death Dis. 2017;8(5):e2790. 28518141 10.1038/cddis.2017.180 PMC5520705

[15] Wang Z , Wang Z , Li G , et al. CXCL1 from tumor‐associated lymphatic endothelial cells drives gastric cancer cell into lymphatic system via activating integrin beta1/FAK/AKT signaling. Cancer Lett. 2017;385:28‐38. 27832972 10.1016/j.canlet.2016.10.043

[16] Nieto MA , Huang RY , Jackson RA , Thiery JP . Emt: 2016. Cell. 2016;166(1):21‐45. 27368099 10.1016/j.cell.2016.06.028

[17] Thiery JP , Acloque H , Huang RY , Nieto MA . Epithelial‐mesenchymal transitions in development and disease. Cell. 2009;139(5):871‐890. 19945376 10.1016/j.cell.2009.11.007

[18] Ozawa T , Riester M , Cheng YK , et al. Most human non‐GCIMP glioblastoma subtypes evolve from a common proneural‐like precursor glioma. Cancer Cell. 2014;26(2):288‐300. 25117714 10.1016/j.ccr.2014.06.005 PMC4143139

[19] Meel MH , Schaper SA , Kaspers GJL , Hulleman E . Signaling pathways and mesenchymal transition in pediatric high‐grade glioma. Cell Mol Life Sci. 2018;75(5):871‐887. 29164272 10.1007/s00018-017-2714-7 PMC5809527

[20] McDonald JT , Gao X , Steber C , et al. Host mediated inflammatory influence on glioblastoma multiforme recurrence following high‐dose ionizing radiation. PLoS One. 2017;12(5):e0178155. 28542439 10.1371/journal.pone.0178155 PMC5439715

[21] Mao P , Joshi K , Li J , et al. Mesenchymal glioma stem cells are maintained by activated glycolytic metabolism involving aldehyde dehydrogenase 1A3. Proc Natl Acad Sci USA. 2013;110(21):8644‐8649. 23650391 10.1073/pnas.1221478110 PMC3666732

[22] Ritchie ME , Phipson B , Wu D , et al. limma powers differential expression analyses for RNA‐sequencing and microarray studies. Nucleic Acids Res. 2015;43(7):e47. 25605792 10.1093/nar/gkv007 PMC4402510

[23] Alafate W , Zuo J , Deng Z , et al. Combined elevation of AURKB and UBE2C predicts severe outcomes and therapy resistance in glioma. Pathol Res Pract. 2019;215(10):152557. 31353228 10.1016/j.prp.2019.152557

[24] Wang J , Cheng P , Pavlyukov MS , et al. Targeting NEK2 attenuates glioblastoma growth and radioresistance by destabilizing histone methyltransferase EZH2. J Clin Invest. 2017;127(8):3075‐3089. 28737508 10.1172/JCI89092 PMC5531394

[25] Wang J , Zuo J , Wahafu A , Wang MD , Li RC , Xie WF . Combined elevation of TRIB2 and MAP3K1 indicates poor prognosis and chemoresistance to temozolomide in glioblastoma. CNS Neurosci Ther. 2019;1‐12. 10.1111/cns.13197 PMC7053231 31318172

[26] Subramanian A , Tamayo P , Mootha VK , et al. Gene set enrichment analysis: a knowledge‐based approach for interpreting genome‐wide expression profiles. Proc Natl Acad Sci USA. 2005;102(43):15545‐15550. 16199517 10.1073/pnas.0506580102 PMC1239896

[27] Bhat KPL , Balasubramaniyan V , Vaillant B , et al. Mesenchymal differentiation mediated by NF‐kappaB promotes radiation resistance in glioblastoma. Cancer Cell. 2013;24(3):331‐346. 23993863 10.1016/j.ccr.2013.08.001 PMC3817560

[28] Phillips HS , Kharbanda S , Chen R , et al. Molecular subclasses of high‐grade glioma predict prognosis, delineate a pattern of disease progression, and resemble stages in neurogenesis. Cancer Cell. 2006;9(3):157‐173. 16530701 10.1016/j.ccr.2006.02.019

[29] Iser IC , Pereira MB , Lenz G , Wink MR . The epithelial‐to‐mesenchymal transition‐like process in glioblastoma: an updated systematic review and in silico investigation. Med Res Rev. 2017;37(2):271‐313. 27617697 10.1002/med.21408

[30] Moreno M , Pedrosa L , Pare L , et al. GPR56/ADGRG1 inhibits mesenchymal differentiation and radioresistance in glioblastoma. Cell Rep. 2017;21(8):2183‐2197. 29166609 10.1016/j.celrep.2017.10.083

[31] Yamini B . NF‐kappaB, mesenchymal differentiation and glioblastoma. Cells. 2018;7(9):125 10.3390/cells7090125 PMC6162779 30200302

[32] Linkous A , Balamatsias D , Snuderl M , et al. Modeling patient‐derived glioblastoma with cerebral organoids. Cell Rep. 2019;26(12):3203‐3211.e5. 30893594 10.1016/j.celrep.2019.02.063 PMC6625753

[33] Yu H , Zhang S , Ibrahim AN , Wang J , Deng Z , Wang M . RCC2 promotes proliferation and radio‐resistance in glioblastoma via activating transcription of DNMT1. Biochem Biophys Res Commun. 2019;516(3):999‐1006. 31277942 10.1016/j.bbrc.2019.06.097

[34] Erstad DJ , Cusack JC Jr . Targeting the NF‐kappaB pathway in cancer therapy. Surg Oncol Clin N Am. 2013;22(4):705‐746. 24012396 10.1016/j.soc.2013.06.011

[35] Yang Y , Zhou H , Zhang G , Xue X . Targeting the canonical Wnt/beta‐catenin pathway in cancer radioresistance: updates on the molecular mechanisms. J Cancer Res Ther. 2019;15(2):272‐277. 30964097 10.4103/jcrt.JCRT_421_18

[36] Iida N , Grotendorst GR . Cloning and sequencing of a new gro transcript from activated human monocytes: expression in leukocytes and wound tissue. Mol Cell Biol. 1990;10(10):5596‐5599. 2078213 10.1128/mcb.10.10.5596-5599.1990 PMC361282

[37] Becker S , Quay J , Koren HS , Haskill JS . Constitutive and stimulated MCP‐1, GRO alpha, beta, and gamma expression in human airway epithelium and bronchoalveolar macrophages. Am J Physiol. 1994;266(3 Pt 1):L278‐L286. 8166297 10.1152/ajplung.1994.266.3.L278

[38] Seifert L , Werba G , Tiwari S , et al. The necrosome promotes pancreatic oncogenesis via CXCL1 and Mincle‐induced immune suppression. Nature. 2016;532(7598):245‐249. 27049944 10.1038/nature17403 PMC4833566

[39] Zhou Y , Zhang J , Liu Q , et al. The chemokine GRO‐alpha (CXCL1) confers increased tumorigenicity to glioma cells. Carcinogenesis. 2005;26(12):2058‐2068. 16033775 10.1093/carcin/bgi182

[40] Bharti AC , Aggarwal BB . Nuclear factor‐kappa B and cancer: its role in prevention and therapy. Biochem Pharmacol. 2002;64(5–6):883‐888. 12213582 10.1016/s0006-2952(02)01154-1

[41] Veeraraghavan J , Natarajan M , Aravindan S , Herman TS , Aravindan N . Radiation‐triggered tumor necrosis factor (TNF) alpha‐NFkappaB cross‐signaling favors survival advantage in human neuroblastoma cells. J Biol Chem. 2011;286(24):21588‐21600. 21527635 10.1074/jbc.M110.193755 PMC3122217

[42] Kesanakurti D , Chetty C , Rajasekhar Maddirela D , Gujrati M , Rao JS . Essential role of cooperative NF‐kappaB and Stat3 recruitment to ICAM‐1 intronic consensus elements in the regulation of radiation‐induced invasion and migration in glioma. Oncogene. 2013;32(43):5144‐5155. 23178493 10.1038/onc.2012.546 PMC3664652

[43] Roos WP , Kaina B . DNA damage‐induced cell death: from specific DNA lesions to the DNA damage response and apoptosis. Cancer Lett. 2013;332(2):237‐248. 22261329 10.1016/j.canlet.2012.01.007

[44] Bakkenist CJ , Kastan MB . DNA damage activates ATM through intermolecular autophosphorylation and dimer dissociation. Nature. 2003;421(6922):499‐506. 12556884 10.1038/nature01368

[45] Pordanjani SM , Hosseinimehr SJ . The role of NF‐kB inhibitors in cell response to radiation. Curr Med Chem. 2016;23(34):3951‐3963. 27554808 10.2174/0929867323666160824162718

[46] Lamouille S , Xu J , Derynck R . Molecular mechanisms of epithelial‐mesenchymal transition. Nat Rev Mol Cell Biol. 2014;15(3):178‐196. 24556840 10.1038/nrm3758 PMC4240281

[47] Carro MS , Lim WK , Alvarez MJ , et al. The transcriptional network for mesenchymal transformation of brain tumours. Nature. 2010;463(7279):318‐325. 20032975 10.1038/nature08712 PMC4011561

[48] Chen MF , Lin CT , Chen WC , et al. The sensitivity of human mesenchymal stem cells to ionizing radiation. Int J Radiat Oncol Biol Phys. 2006;66(1):244‐253. 16839703 10.1016/j.ijrobp.2006.03.062

[49] Clavin NW , Fernandez J , Schonmeyr BH , Soares MA , Mehrara BJ . Fractionated doses of ionizing radiation confer protection to mesenchymal stem cell pluripotency. Plast Reconstr Surg. 2008;122(3):739‐748. 18766036 10.1097/PRS.0b013e318180edaa

[50] Tomuleasa C , Soritau O , Brie I , et al. Mesenchymal stem cell irradiation in culture engages differential effect of hyper‐fractionated radiotherapy for head and neck cancers. J BUON. 2010;15(2):348‐356. 20658734

[51] Chua HL , Bhat‐Nakshatri P , Clare SE , Morimiya A , Badve S , Nakshatri H . NF‐kappaB represses E‐cadherin expression and enhances epithelial to mesenchymal transition of mammary epithelial cells: potential involvement of ZEB‐1 and ZEB‐2. Oncogene. 2007;26(5):711‐724. 16862183 10.1038/sj.onc.1209808

[52] Edwards LA , Woolard K , Son MJ , et al. Effect of brain‐ and tumor‐derived connective tissue growth factor on glioma invasion. J Natl Cancer Inst. 2011;103(15):1162‐1178. 21771732 10.1093/jnci/djr224 PMC3149042

