# Supplementary figures and images for: Elevation of CXCL1 indicates poor prognosis and radioresistance by inducing mesenchymal transition in glioblastoma
Source: CNS Neurosci Ther. 2020 Mar 18;26(4):475–85. doi: 10.1111/cns.13297 (PMC7080429; doi:10.1111/cns.13297)

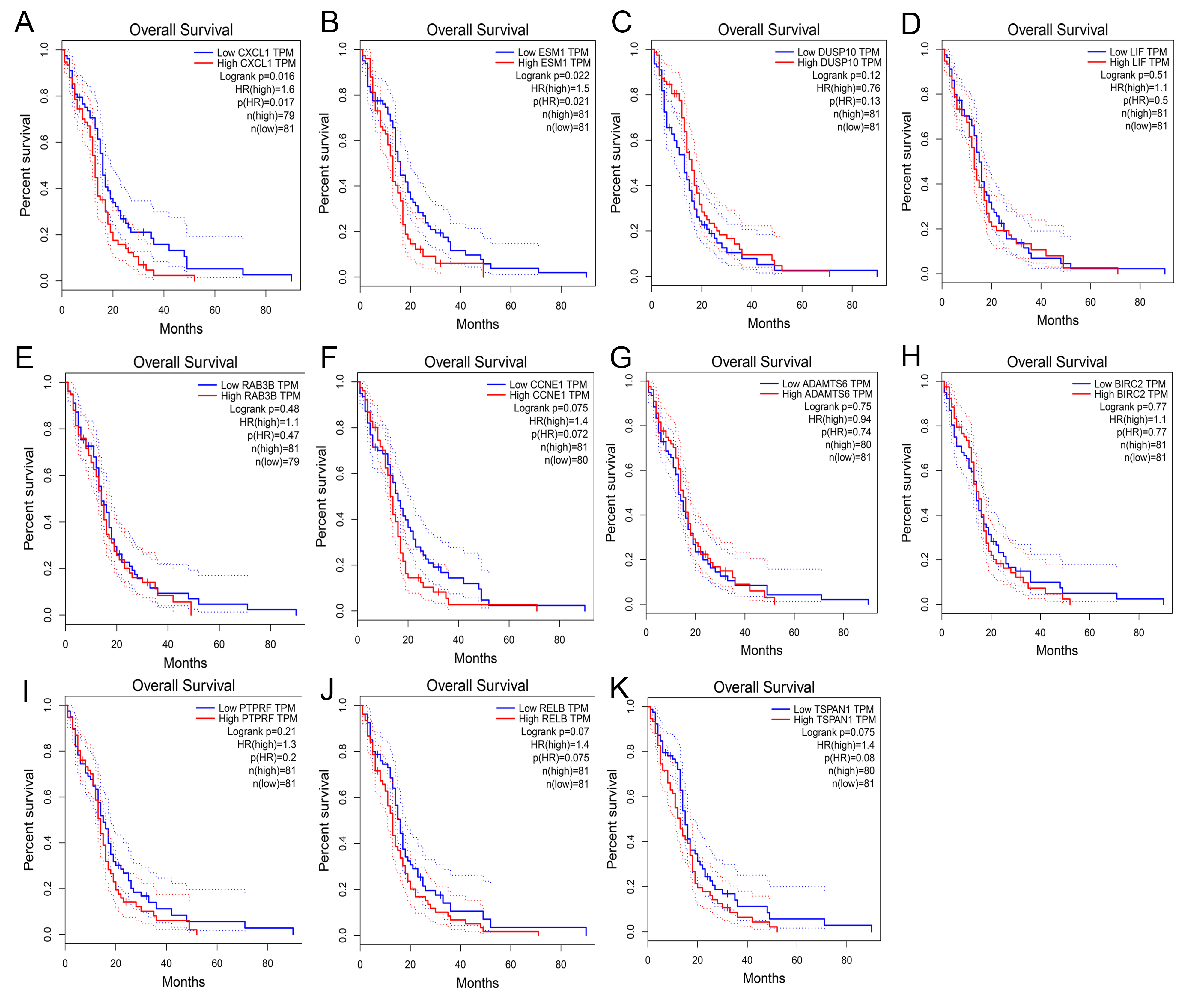

Supplement: Supplementary file 1 [file CNS-26-475-s001.tif]
